# Improved reproductive performance achieved in tropical dairy cows by dietary beta-carotene supplementation

**DOI:** 10.1038/s41598-021-02655-8

**Published:** 2021-11-30

**Authors:** Soparak Khemarach, Saowaluck Yammuen-art, Veerasak Punyapornwithaya, Sutichai Nithithanasilp, Narongrit Jaipolsaen, Siwat Sangsritavong

**Affiliations:** 1 Department of Animal and Aquatic Science, Faculty of Agriculture, Chiang Mai University, Chiang Mai, 50200 Thailand; 2 Department of Food Animal Clinics, Faculty of Veterinary Medicine, Chiang Mai University, Chiang Mai, 50100 Thailand; 3 National Center for Genetic Engineering and Biotechnology, Thailand Science Park, 12120 Thailand

**Keywords:** Biotechnology, Physiology

## Abstract

Dairy farming in tropical climates is challenging as heat stress can impair reproduction in cows. Previous studies have demonstrated the beneficial effects of beta-carotene supplementation on bovine reproductive performance. This study was performed in Thailand, where the temperature-humidity index (THI) during the experimental periods was measured to range from 78.4 to 86.1. Lactating Holstein cows classified as repeat breeders (previous artificial insemination [AI] failures) were randomly assigned into two treatments, control treatment (T1; received placebo, n = 200) and test treatment (T2; received 400 mg/h/day of beta*-*carotene, n = 200). All cows were subjected to a protocol for synchronization of ovulation and timed artificial insemination (TAI). The day of the 1st ovulation synchronized protocol was defined as day 0, and the total experimental period was 160 days. Daily placebo or beta-carotene supplements were given orally on day 0 and each subsequent day of the experiment. Diagnosis of pregnancy was performed using ultrasound on day 30 after insemination. Non-pregnant cows were subjected to further ovulation synchronizations (maximum of four) and TAI over a period of 160 days. Milk samples were collected every ten days throughout the experiment. The samples were analyzed for beta-carotene concentration, superoxide dismutase (SOD) and glutathione peroxidase (GPx) activities. The pregnancies per AI of the cows in T2 were significantly greater than that of T1 from the 2nd to 4th TAI. During the entire experimental period, the pregnancies in T2 were significantly greater than that of T1. Cox's proportional hazards regression model data indicated a 44% greater probability of pregnancy for cows receiving beta-carotene. The concentrations of milk beta-carotene in T2 were significantly greater than T1 from the 2nd to 4th TAI. Significantly greater SOD and GPx activities were observed in T2 than T1, suggesting a reduction of oxidative stress in cows treated with beta-carotene. Dietary supplementation with beta-carotene thus improves the reproductive performance of repeat breeders exposed to heat stress, possibly by reducing oxidative stress.

## Introduction

Bovine reproductive disorders are major problems in the dairy industry^1^, which can incur economic loss^2,3^. Estimated losses from reproductive disorders in U.S. dairy farms amount to $70 per cow per year when the days open extended to 130 days and $220 per cow per year with average days open beyond 160 days^4^. Simple and cost-effective strategies are needed to improve reproductive performance in the dairy industry. Micronutrient supplementation, in particular beta-carotene, is a proven strategy for ameliorating reproductive disorders in cows. For instance, Arechiga et al.^5^ and De Ondarza et al.^6^ reported increased conception rates in lactating cows orally supplemented with beta-carotene, and beta-carotene injections were shown to improve reproductive performance in lactating dairy cows^7^. Moreover, lactating cows with higher circulating beta-carotene on the day of artificial insemination (AI) show greater conception rates together with a lower incidence of pregnancy loss^8^.

The beneficial effects of beta-carotene in lactating cows are ascribed to its known roles. One major role is defending against oxidative stress (OS), including quenching of singlet oxygen and scavenging peroxyl radicals in the oocyte and embryo^5,9^, and deactivating reactive oxygen species (ROS) from cholesterol cleavage enzyme^10^. ROS defense is mediated by intracellular beta-carotene in follicles and oocytes and extracellular beta-carotene in follicular fluid^11^. Beta-carotene is also known to enhance oocyte maturation^11^, and beta-carotene supplementation has been reported to increase circulating hydroxyproline^12^, the crucial regulator of placental development^13,14^ and fetal nutrition^14^. Furthermore, beta-carotene supplementation has been reported to reduce neutrophils in smears taken from the uterus, which is a marker of good uterine health^12^.

The beneficial effects of beta-carotene supplementation on reproductive performance are more marked when lactating cows are exposed to heat stress^5^. Seasonal heat stress can reduce conception rates by ten percentage points^15^. Therefore, beta-carotene supplementation could be particularly important for dairy farming in tropical areas such as Thailand, where the temperature-humidity index (THI) ranges from 72 to 89^12^, which exceeds the dairy cow comfort threshold of 71^16^. Previous studies have demonstrated benefits from dietary supplements of beta-carotene from natural sources, such as dried Leucaena leaves (*Leucaena leucocepphala*) and dried Marigold flower (*Tagetes erecta *L.), which have been shown to increase the fertility of lactating dairy cows raised in Thailand^17,18^. However, the beneficial effects of beta-carotene derived from natural products are difficult to quantify because of their high variation of beta-carotene contents.

The minimum circulating level of beta-carotene conducive for reproduction in cows is 3–4 µg/mL^19,20^, whereas increased plasma concentrations of beta-carotene (4–5 µg/mL) can lead to even greater reproductive efficiency^8^. A study of dairy cows raised in northern Thailand that had received corn silage as the major source of roughage reported circulating plasma beta-carotene concentrations of 1.36–1.88 µg/mL^17^, indicating a deficiency of beta-carotene. The deficiency of beta-carotene, together with the effects of heat stress, could be responsible for the subfertility observed in dairy cows raised in Thailand. Thus, we hypothesized that continuous beta-carotene supplementation (400 mg/h/day) would increase the reproductive efficiency of dairy cows classified as repeat breeders (previous AI failures) exposed to heat stress incidental to dairy farming in a tropical climate.

## Methods

### Ethics approval

The Institutional Animal Care and Use Committee, National Center for Genetic Engineering and Biotechnology, Thailand, has approved this research project by the Ethical Principles for the Use of Animals for Scientific Purposes issued by the National Research Council of Thailand. The approval for the Care and Use of Animals for Scientific Purposes Code BT-Animal 17/2561 was granted in February 2018. We confirm that all methods were carried out under relevant guidelines and regulations. Additionally, this work complied with the essential ARRIVE guidelines recommended by the National Centre for the Replacement, Refinement, and Reduction of Animals in Research (London, UK).

### Animal and management

The study was carried out in commercial dairy farms (30 farms) located in central Thailand (14° 52′ 48.5″ N,101° 16′ 24.4″ E). The average THI, calculated from the well-adopted equation^21^, during this experiment was 82.1 ± 5.1 (s.d = 5.1, range 78.4–86.1). The study comprised 400 lactating Holstein Friesian cows, housed in a loose-housing system, and the number of cows in each farm is shown in Supplementary Table 1. Only cows with reproductive problems, i.e., cows that had received three or more services but were not pregnant (the average number of previous AI was 4.1 ± 1.5) were enrolled. These repeat breeders in each particular farm were randomly divided into two treatments. The control treatment (T1; n = 200) received a placebo (EMPTY GELATIN CAPSULE; Unicommerce4u Co., Ltd., Bangkok, Thailand) daily while the cows assigned to test treatment (T2; n = 200) received 400 mg/h/day of beta-carotene (LUCAROTIN 10% β-CAROTENE; BASF SE, Ludwigshafen, Germany). Both capsules (control or test treatment) were given to cows during the morning feeding session. Supplementation started on day 0 and was continued daily until the end of the experiment. Six student trainees were responsible for giving the capsules to the cows and also collecting the milk samples.

The cows in both T1 and T2 treatments received similar total mixed rations (TMR) ad libitum, and clean drinking water was available at all times. Corn silage was the main source of roughage in TMR.

### Ovulation synchronization protocol

**Figure 1:**
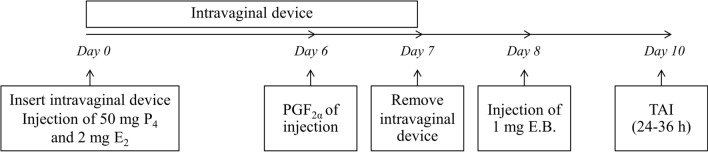
The synchronization protocol. Intravaginal device (CIDR, DEC International NZ Limited, Hamilton, New Zealand), PGFα (JURAMATE, Jurox Pty. Limited, NSW, Australia), progesterone (P_4_), estradiol 17 β (E_2_) and estradiol benzoate (EB) (Steraloids Inc., Newport, RI, USA).

All cows were subjected to a protocol for synchronization of ovulation and timed artificial insemination (TAI)^22^ (Fig. 1). The factors potentially affecting reproductive performance, including farms, days in milk, parities, body condition score, milk yield and sires, were included in the statistical model. Frozen semen from two bulls (ALTADISCO AND ALTADURST; Alta Genetics Inc, Alberta, Canada) was used in this experiment. Before the inseminations, semen quality assessment was performed by computer-assisted sperm analyzers (CASA).

**Figure 2:**
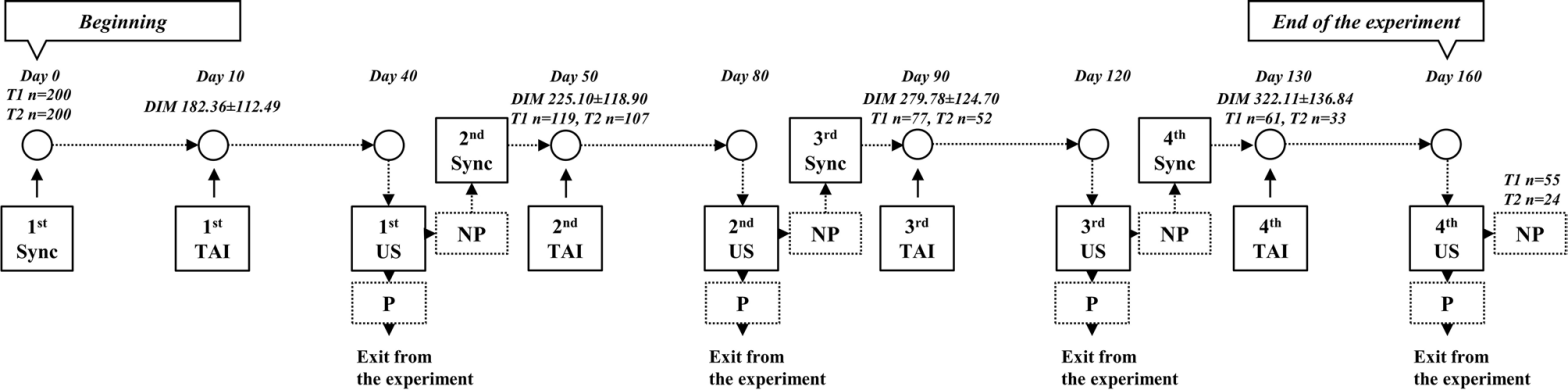
The experimental design. Cows were given a placebo or 400 mg/h/day of beta-carotene daily, from the 1st synchronization (day 0) until the experiment ended. *Sync* synchronization, *TAI* timed artificial insemination, *P* pregnant, *NP* non-pregnant, *US* ultrasound evaluation.

Diagnosis of pregnancy was performed with ultrasound (HS-1600 V, Honda Electronics Co., Ltd., Toyohashi, Japan) 30 days after insemination. Non-pregnant cows were subjected to the second and subsequent synchronization protocols up to a maximum of four synchronizations. Pregnant cows were immediately released from the experiment (Fig. 2).

### Data and sample collections

Feed samples were taken every 30 days; thus, six feed samples were taken from each farm (a total of 180 samples) and kept at − 20 °C for analyses. Since plasma and milk biomarkers are positively correlated^23,24^, we measured all biomarkers of interest in milk instead of plasma. Milk samples were collected every ten days throughout the experiment, including the same days that TAI was carried out. Samples collected at TAI (day 10, 50, 90 and 130) were used to compare T1 and T2. Samples collected on the other days preceding each TAI were used as covariates, as shown in the statistical model. Milk samples were preserved with sodium azide^25^ (THEMOFISHER SCIENTIFIC AUSTRALIA Pty Ltd., NSW, Australia) and kept at 4 °C prior to measurements of beta-carotene concentrations, and activities of SOD and GPx.

### Analyses of the samples

All of the 180 feed samples (from 30 farms sampled at six-time points) were evaluated for chemical composition by the following tests: AOAC^26^; dry matter (DM); Method 934.01, crude protein (CP); Method 968.06 and ether extract (EE); Method 920.39. Detergent methods^27^ were employed to determine fiber content (neutral detergent fiber (NDF), acid detergent fiber (ADF) and acid detergent lignin (ADL). Beta-carotene concentrations in feed were measured using HPLC (WATER, Milford, MA, USA) as described by Tee and Lim^28^ and beta-carotene concentrations in milk samples were measured as described by Plozza et al.^29^. The % CV for intra- and inter-assays of beta-carotene was 8.51% and 9.27%, respectively. The SOD activity in milk samples was measured using a spectrophotometer as described by Gao et al.^30^, in which % CV for intra- and inter-assays were 9.92% and 12.21%, respectively. The GPx activity was measured according to the method described by Torres et al.^31^, in which % CV for intra- and inter-assays were 8.92% and 11.29%, respectively.

### Statistical analyses

All statistical analyses were performed using the R program environment (Version R-3.6.1). The effects of treatment (T1 and T2) on pregnancy per AI at 10 (1st TAI), 50 (2nd TAI), 90 (3rd TAI) and 130 (4th TAI) days were analyzed using a generalized linear mixed model with logit link function or a mixed-effect logistic regression model. The model was designed as a binomial logit model with fixed effects of treatment (treatments; 1 = T1 and 2 = T2) on pregnancy status (non-pregnant = 0, pregnant = 1), and covariate independent variables including parity (par = 1, 2, 3, and 4), Body Condition Score (BCS = 2.50, 2.75, and 3.00) and sires (sires = 1 and 2). Farm (farm = 1, 2, 3, …30) was defined as a random effect in the model. The link function is defined as follows:

$$g\left(\mu \right)=\mathrm{ln}\left(\frac{{\varvec{p}}}{1-{\varvec{p}}}\right)=\mathrm{logit }\left(p\right).$$

Thus, the generalized linear mixed model with logit link function is as follows.

$$\mathrm{ln}\left(\frac{{p}_{i}}{1-{p}_{i}}\right)={\beta }_{0}+{\beta }_{1}{x}_{1}+{\beta }_{2}{x}_{2}+{\beta }_{3}{x}_{3}+{\beta }_{4}{x}_{4}+{farm}_{j},$$

where $p_{i}$ is the probability of pregnancy $i$th from the $j{\text{th}}$ farm, $\beta_{0}$ is an intercept, $x_{1}$ is the fixed effects from treatment, $x_{2}$ is the fixed effects from parity, $x_{3}$ is the fixed effects from BCS, $x_{4}$ is the fixed effects from sires, $\beta_{1} ,\beta_{2} ,\beta_{3} {\text{ and }} \beta_{4}$ the estimated coefficients corresponding to $x_{1} ,x_{2} ,x_{3}$*,*$x_{4}$*,* $farm_{j}$ is the random effect from $j{\text{th}}$ farm.

Kaplan–Meyer survival curves were constructed to test for differences in median non-pregnant days between the cows in T1 and T2 (uncensored case = pregnant; censored = non-pregnant); log-rank test was used to compare survival curves.

Effects of treatment on pregnancy rate during 0–160 days were analyzed using Cox's proportional hazards regression model. The model included effects of treatments (treatments; 1 = T1 and 2 = T2), parity (par = 1, 2, 3, and 4), BCS (BCS = 2.50, 2.75, and 3.00), farm (farm = 1, 2, 3, …30) and sires (sires = 1 and 2) as categorical. Whereas days in milk (DIM) was included as a continuous variable. The model was written as:

$$h\left(t\right)={h}_{0}\left(t\right)\times \mathrm{exp}\left({\beta }_{1}treatment+{\beta }_{2}par+{\beta }_{3}BCS+{\beta }_{4}farm+{\beta }_{5}DIM+{\beta }_{6}sires\right),$$

where $h(t)$ is the hazard function; ${h}_{0}(t)$ is the baseline hazard at time $t$; $t$ is the survival time; ${\beta }_{1},...,{\beta }_{6}$ is the coefficients of variables in the model; *treatment*, *par, BCS*, *farm*, *DIM* and *sires* is the fixed effect variables. The assumptions of the proportional hazards, nonlinearity and influential observation were tested by examining Schoenfeld residuals, Martingale and Deviance residuals, respectively.

Effects of beta-carotene supplementation on SOD and GPx activity in milk were analyzed using a generalized linear mixed model (GLMM). The differences in means of beta-carotene, SOD and GPx activity between T1 and T2 at day 0 were analyzed using the following model:

$${y}_{ir}=\mu +{treatment}_{i}+co{w}_{r}+{farm}_{k}+{treatment}_{i}\times {farm}_{k}+\boldsymbol{ }{\varepsilon }_{irk},$$

where ${y}_{ir}$ is the observation from *r*th dairy cow from *i*th treatments; $\mu$ is the overall mean; ${treatment}_{i}$ is the effects of *i*th treatments *(*$i$ = 1 and 2; 1 = T1 and 2 = T2*)*; $co{w}_{r}$ is the random effects from an individual *r*th cow; ${farm}_{k}$ is the random effects from individual *k*th farm**,** ${treatment}_{i}\times {farm}_{k}$ is the interaction random effect from treatment and farm, and ${\varepsilon }_{irk}$ is the random error.

Furthermore, the differences in means of beta-carotene, SOD and GPx activity between T1 and T2 were analyzed at 10 (1st TAI), 50 (2nd TAI), 90 (3rd TAI) and 130 (4th TAI) days. For the statistical model, the average beta-carotene and enzyme activity parameters (i.e., SOD and GPx) at day 0 and the period of day 0 to day 40, day 0 to day 80, day 0 to day 120 were included in the model for 1st, 2nd, 3rd and 4th TAI respectively. The GLMM was given by:

$${y}_{ijr}=\mu +{treatment}_{i}+\beta \left({x}_{j}\right)+co{w}_{r}+{farm}_{{\varvec{k}}}+{treatment}_{i}\times {farm}_{k}+{\varepsilon }_{ijkr}$$

where ${y}_{ijr}$ is the observation from *r*th dairy cow from *i*th treatment having *j*th period as a covariate; $\mu$ is the overall mean; ${treatment}_{i}$ is the effects of *i*th treatment *(*$i$ = 1 and 2; 1 = T1 and 2 = T2*)*; $\beta ({x}_{j})$ is the average of beta-carotene, SOD and GPx from *j*th period; $co{w}_{r}$ is the random effects from an individual *r*th cow; ${farm}_{k}$ is the random effects from the individual *k*th farm, ${treatment}_{i}\times {farm}_{k}$ is the interaction random effect from treatments and farm, and ${\varepsilon }_{ijkr}$ is the random error*.*

Assumptions, including normality and homogeneity of variance of residuals, were examined using the Q–Q normality plot and the plot between residuals and fitted values, respectively. In all tests conducted, *P-*values lower than 0.05 were considered significant.

## Results

Chemical Compositions of the TMR given during the experiment were: DM 35.6 ± 3.2% (range 34.0–37.3%), CP 16.6 ± 2.5% (range 14.9–18.6%), EE 4.3 ± 1.4% (range 3.1–5.2%), NDF 38.2 ± 3.1% (range 34.5–39.3%), ADF 22.4 ± 2.3% (range 20.4–24.7%), ADL 11.3 ± 3.8% (range 10.1–14.7%) and beta-carotene 17.0 ± 2.5 mg/kgDM (range 15.2–19.3 mg/kgDM). Average DIM at the beginning of the experiment was 203 ± 90 days in T1 and 205 ± 97 in T2 (*P* = 0.09), whereas the average parities were 2.8 ± 1.3 and 2.9 ± 1.3 in T1 and T2 (*P* = 0.26), respectively. The average BCS in T1 and T2 cows was 2.7 ± 1.3 and 2.8 ± 1.5 (*P* = 0.90) at the beginning of the experiment. The average BCS was 2.6 ± 1.2 and 2.7 ± 1.1 in T1 and T2 at the end of the experiment (*P* = 0.23). At the beginning of the experiment, the average milk yield was 15.6 ± 3.6 and 15.5 ± 4.6 kg/h/day in T1 and T2 (*P* = 0.85). Milk production declined slightly at the end of the experiment (13.8 ± 3.6 vs. 14.7 ± 3.6 kg/h/day; in T1 and T2 (*P* = 0.93)).

The pregnancy per AI at the first insemination did not differ (*P* = 0.26) between treatments (T1 = 40.70 ± 0.04 vs. T2 = 47.40 ± 0.04). However, the pregnancy per AI of cows in T2 was greater than that in T1 in all subsequent inseminations (Table 1).

**Table 1:** Pregnancy per AI (LSM ± SE) of control treatment (T1, n = 200) and test treatment (T2, n = 200). Data in parentheses indicate the numbers of pregnant cows (numerator) and cows at risk (denominator).

Synchronization	T1	T2	*P*-value
1st TAI	40.70 ± 0.04 (81/200)	47.40 ± 0.04 (93/200)	0.26
2nd TAI	34.80 ± 0.05 (42/119)	50.60 ± 0.05 (55/107)	0.04
3rd TAI	18.70 ± 0.05 (16/77)	34.90 ± 0.07 (19/52)	0.04
4th TAI	9.49 ± 0.03 (6/61)	22.44 ± 0.11 (9/33)	0.04
Total pregnancies	74.50 ± 0.03 (145/200)	89.80 ± 0.02 (176/200)	< 0.01

**Figure 3:**
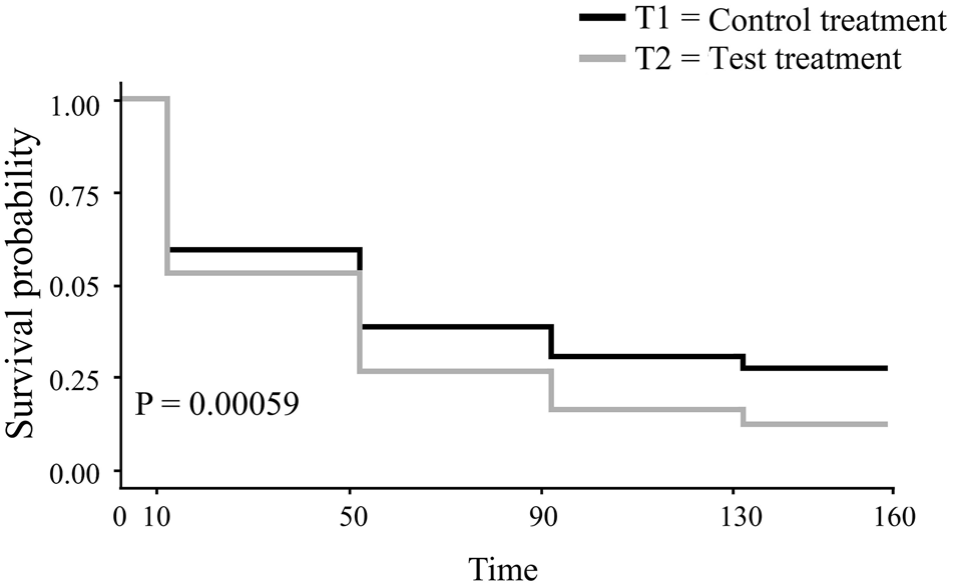
The survival curve of non-pregnancy. The figure was generated from the Kaplan–Meyer survival curve. Categorical variables included treatment, parity, body condition score, sires and farm, whereas days in milk were included as a continuous variable. T1 = Control treatment, T2 = Test treatment.

The analysis using Cox's proportional hazards regression model indicated that cows receiving beta-carotene had a significantly greater probability of pregnancy per AI. In contrast, parities, BCS, DIM, farms, and sires were not significant (Table 2). The non-pregnancy survival curve is shown in Fig. 3.

**Table 2:** Effects of treatment on the likelihood of pregnancy in dairy cows with and without beta-carotene. Parity and body condition score (BCS) were included as covariate variables. Days in milk and sires were also included in the model as stratified variables.

Factor	Parameter estimate	Standard error	Hazard ratio	*P*-value
Treatment	Reference class			
	0.37	0.02	1.44	< 0.01
Parity	Reference class			
	− 0.06	0.04	0.95	0.25
BCS	Reference class			
	0.13	0.01	1.14	0.68

**Figure 4:**
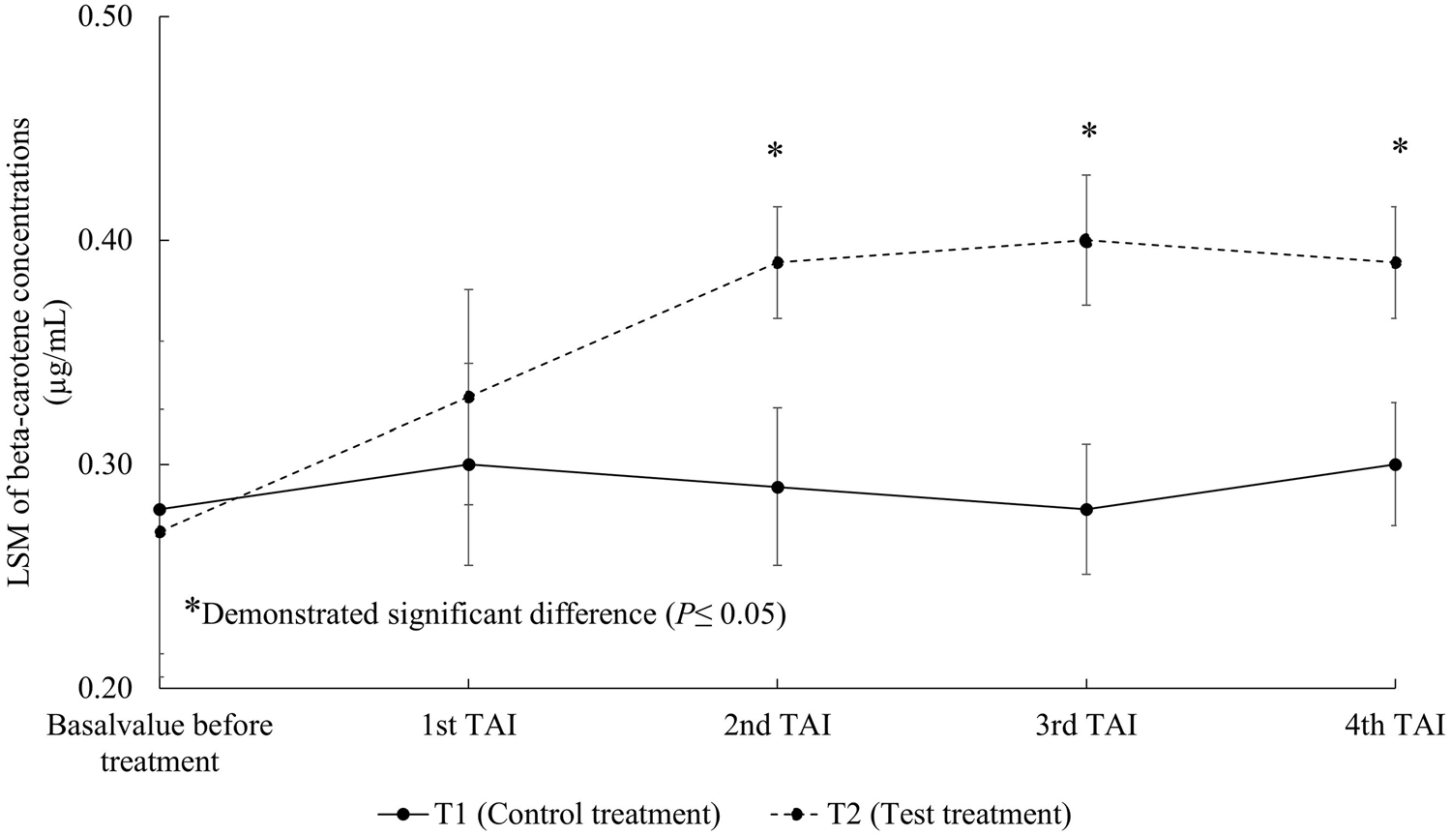
Least-squares means (LSM) of beta-carotene concentrations (µg/mL) in milk at the same time of each timed artificial insemination (TAI). T1 = Control treatment, T2 = Test treatment.

The basal concentrations of beta-carotene in milk between T1 and T2 were not significantly different (*P* = 0.56). Although the milk beta-carotene concentrations in T1 and T2 were not significantly different at the first TAI, the concentration of milk beta-carotene in T2 was significantly greater after each subsequent synchronization (Fig. 4).

**Figure 5:**
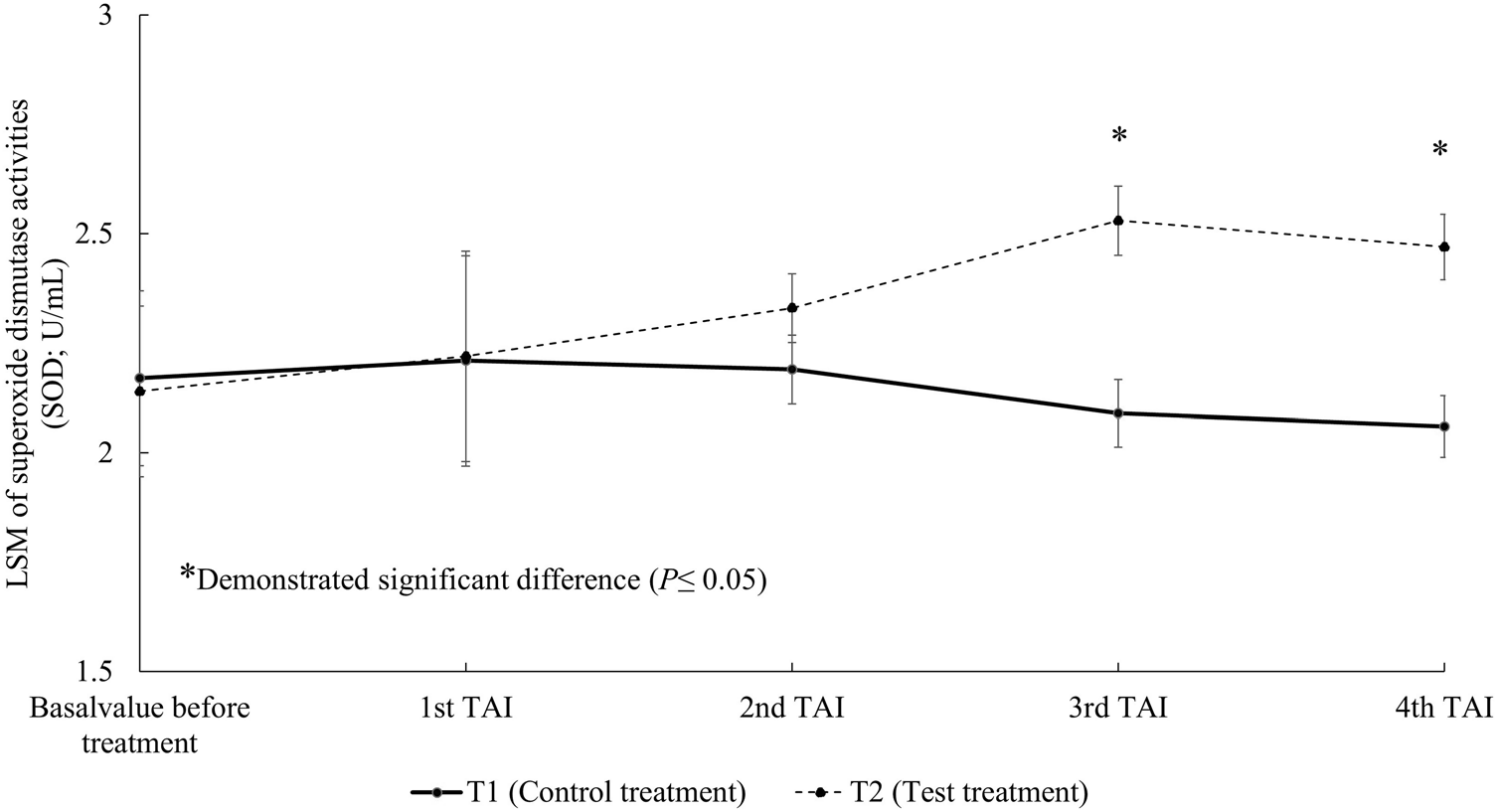
Least-squares means (LSM) of superoxide dismutase activities (SOD; U/mL) in milk at the same time of each timed artificial insemination (TAI). T1 = Control treatment, T2 = Test treatment.

The basal activities of superoxide dismutase (SOD) and the activity at the first and second TAI were not significantly different between T1 and T2. SOD activities were significantly higher in T2 at the third and fourth TAI (Fig. 5).

**Figure 6:**
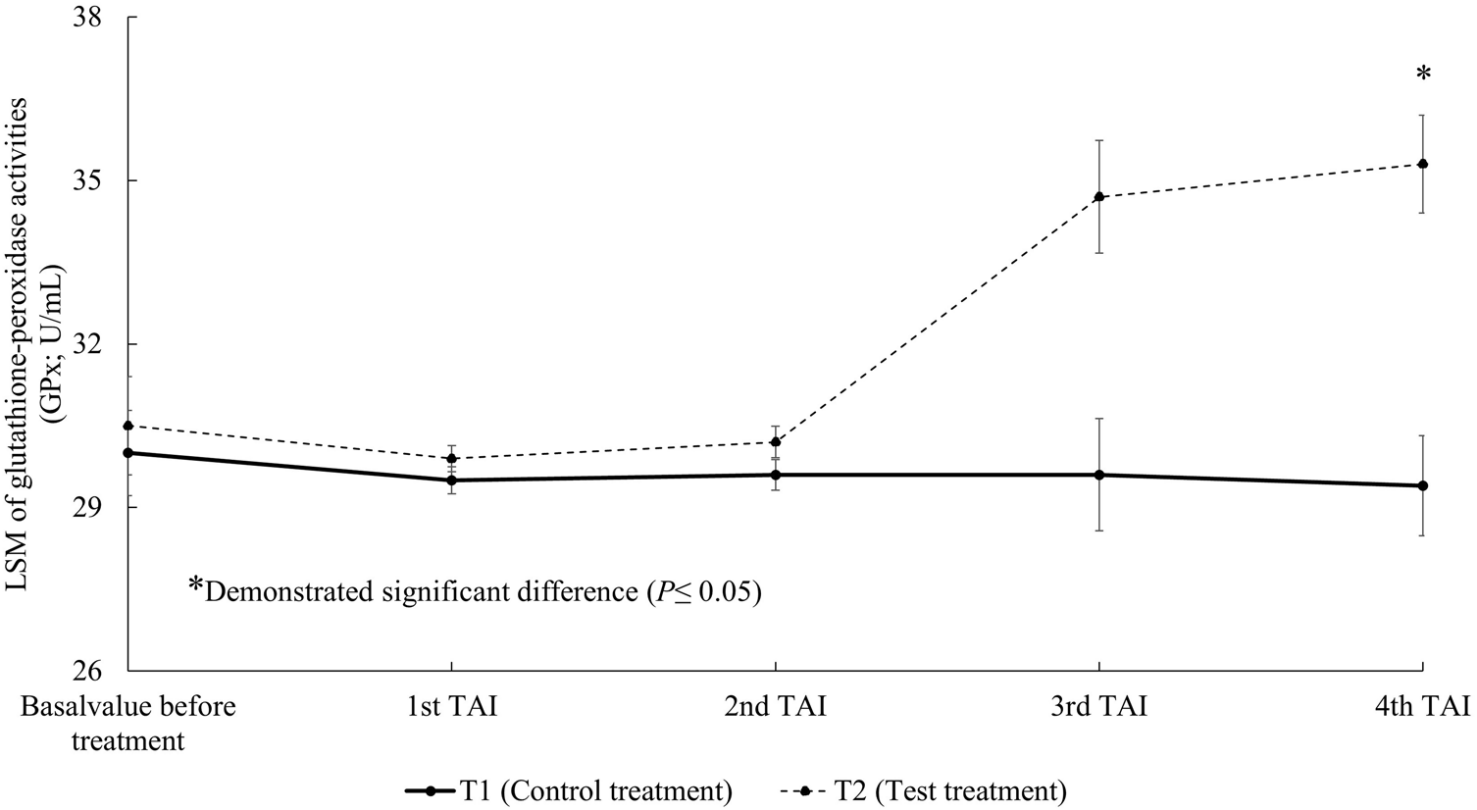
Least-squares means (LSM) of glutathione-peroxidase activities (GPx; U/mL) in milk at the same time of each timed artificial insemination (TAI). T1 = Control treatment, T2 = Test treatment.

The basal activities of glutathione peroxidase (GPx) and at the first, second and third TAI were not significantly different. In contrast, on the fourth TAI, GPx activity in T2 was significantly greater than in T1 (*P* = 0.03) (Fig. 6).

## Discussion

Domestic farm cows raised in tropical climates are subjected to heat stress. Heat stress is known to increase ROS in preimplantation bovine embryos^32^, suggesting that farm cows raised in the tropics are likely to be subjected to OS with a major impact on the mammalian female reproductive process^33^, including early embryonic losses, intrauterine growth restriction and fetal death^34^. We tested the hypothesis that cows raised in the tropics subjected to heat stress and consequent OS would show better reproductive performance when treated with beta-carotene. The T2 showed significantly greater pregnancy per AI after the second and all subsequent synchronizations (Table 1). Moreover, treatment was a significant factor in Cox’s proportional hazard regression for the probability of getting pregnant of the studied cows (Table 2). The T2 showed a significantly different survival curve from T1 (Fig. 3). These results are congruent with the study by Arechiga et al.^5^, which demonstrated significantly increased pregnancy rates in cows treated with beta-carotene when subjected to heat stress. However, other studies with fewer cows (and possibly under-powered) found no significant effect of beta-carotene treatment^35–37^. Adverse effects of beta-carotene treatment were observed when administered at higher doses^38^.

The supplementation of 300 mg/h/day in dairy cows failed to increase plasma beta-carotene concentrations in one study^39^, whereas Arechiga et al.^5^ reported increases of plasma beta-carotene from 3.06 ± 0.23 to 6.00 ± 0.16 µg/mL when 400 mg/h/day of beta-carotene was supplemented, which was accompanied by increased pregnancy rates in the supplemented cows. De Ondarza et al.^6^ reported that the supplementation of 425 mg/h/day trend to increase pregnancy per AI. In our study, the same dose of beta-carotene supplementation (400 mg/h/day) that was shown previously to increase pregnancy rates^5^ was chosen. The concentration of beta-carotene in milk was significantly higher in treated cows from the second TAI onwards (Fig. 4), which was consistent with the observed increased pregnancy per AI at the same measured intervals among cows receiving treatment. The activities of antioxidant enzymes SOD and GPx in milk among cows receiving treatment were also significantly greater than control cows (Figs. 5, 6), suggesting that the elevated beta-carotene concentrations in treated cows led to induction of enzyme activities. We speculated that the increased SOD and GPx activities reduced OS in treated cows that in turn led to cow physiology more conducive to reproduction. The roles of SOD and GPx in mammalian reproduction are well documented. SOD is involved in antral follicle development^40^. Matzuk et al.^41^ reported that SOD knock-out mice showed subfertility as evidenced by fewer preovulatory follicles and corpus lutea found under histological analysis.

Furthermore, SOD activity is positively correlated with intrafollicular estradiol, which is related to oocyte quality^42^. GPx has general cellular functions, including cell proliferation, differentiation, and apoptosis^43^. For reproductive function, GPx increases gamete viability and fertilization^40^. Furthermore, high GPx content in oocytes during follicular development is related to improved development competence of follicles^44–46^. Paszkowski et al.^47^ reported that activities of GPx in the follicular fluid of fertilized follicles were higher than that of fluid from unfertilized follicles. Ovarian GPx may also be involved in follicular development, as the suppression of ovarian GPx resulted in an increased percentage of atretic and apoptotic antral follicles^48^.

## Conclusion

The effect of beta-carotene dietary supplement was tested in repeat breeding lactating Holstein raised in Thailand. Beta-carotene supplementation increased the probability of getting pregnant by 44% (HR = 1.44). The activities of SOD and GPx were increased in the milk of cows receiving supplements. Therefore, increased activities of antioxidant enzymes in cows receiving supplements could lead to a reduction of OS. We speculate that the improvement in reproductive performance is due to reduced ovulation failures, although other factors cannot be ruled out. Beta-carotene dietary supplementation might lead to better reproductive efficiency in repeat breeders, particularly in cows exposed to heat stress in a tropical environment.

## Supplementary Information

Supplementary Information.
