# Supplementary material for: Improved reproductive performance achieved in tropical dairy cows by dietary beta-carotene supplementation
Source: Sci Rep. 2021 Nov 30;11:23171. doi: 10.1038/s41598-021-02655-8 (PMC8633284; doi:10.1038/s41598-021-02655-8)
Supplement: Supplementary file 1 — Supplementary Information. [file 41598_2021_2655_MOESM1_ESM.docx]

| Farm ID | T1 | T2 | total |
| --- | --- | --- | --- |
| 1 | 4 | 4 | 8 |
| 2 | 7 | 7 | 14 |
| 3 | 20 | 20 | 40 |
| 4 | 2 | 2 | 4 |
| 5 | 14 | 14 | 28 |
| 6 | 14 | 14 | 28 |
| 7 | 15 | 14 | 29 |
| 8 | 4 | 4 | 8 |
| 9 | 6 | 6 | 12 |
| 10 | 5 | 5 | 10 |
| 11 | 10 | 10 | 20 |
| 12 | 11 | 11 | 22 |
| 13 | 7 | 7 | 14 |
| 14 | 6 | 5 | 11 |
| 15 | 5 | 6 | 11 |
| 16 | 11 | 11 | 22 |
| 17 | 2 | 2 | 4 |
| 18 | 2 | 2 | 4 |
| 19 | 2 | 2 | 4 |
| 20 | 3 | 3 | 6 |
| 21 | 3 | 3 | 6 |
| 22 | 6 | 6 | 12 |
| 23 | 2 | 2 | 4 |
| 24 | 2 | 2 | 4 |
| 25 | 4 | 4 | 8 |
| 26 | 3 | 4 | 7 |
| 27 | 9 | 9 | 18 |
| 28 | 14 | 14 | 28 |
| 29 | 4 | 4 | 8 |
| 30 | 3 | 3 | 6 |
| total | 200 | 200 | 400 |

**Supplement table 1.** The number of studied animals in each farm (a total of 30 farms; control treatment; T1, n = 200 and test treatment; T2, n = 200).
